# Deciphering genome-wide transcriptomic changes in grapevines heavily infested by spotted lanternflies

**DOI:** 10.3389/finsc.2022.971221

**Published:** 2022-08-25

**Authors:** Md Tariqul Islam, Crosley Kudla-Williams, Suraj Kar, Jason P. Londo, Michela Centinari, Cristina Rosa

**Affiliations:** ^1^ Department of Plant Pathology and Environmental Microbiology, The Pennsylvania State University, University Park, PA, United States; ^2^ Department of Plant Science, The Pennsylvania State University, University Park, PA, United States; ^3^ School of Integrative Plant Science Horticulture Section, Cornell AgriTech, Cornell University, Geneva, NY, United States

**Keywords:** transcriptome, plant-insect interactions, invasive species, spotted lanternfly, grapevine

## Abstract

The spotted lanternfly, a newly invasive insect in the U.S. that is a great concern for the grapevine industry, produces damage on its host plants through aggressive feeding, using a piercing and sucking method to feed on the phloem of plants. In the eastern US, adult SLF can invade vineyards through fruit ripening until the end of the growing season; however, it is still unclear how prolonged late-season SLF feeding can affect the health of grapevines, as well as the host responses to this extensive damage. Thus, we have performed a comprehensive genome-wide transcriptome analysis in grapevines heavily infested by the spotted lanternfly, as it occurs in Pennsylvania vineyards, and compared it to other relevant transcriptomes in grapes with different degrees to susceptibility to similar pests. Among a variety of plant responses, we highlight here a subset of relevant biological pathways that distinguish or are common to the spotted lanternfly and other phloem feeders in grapevine. The molecular interaction between spotted lanternfly and the vine begins with activation of signal transduction cascades mediated mainly by protein kinase genes. It also induces the expression of transcription factors in the nucleus, of other signaling molecules like phytohormones and secondary metabolites, and their downstream target genes responsible for defense and physiological functions, such as detoxification and photosynthesis. Grapevine responses furthermore include the activation of genes for cell wall strengthening *via* biosynthesis of major structural components. With this study, we hope to provide the regulatory network to explain effects that the invasive spotted lanternfly has on grapevine health with the goal to improve its susceptibility.

## 1 Introduction

The spotted lanternfly (SLF), *Lycorma delicatula* (White), is a newly invasive insect of the U.S (1). Native to Asia, the first report of SLF being found in the United States was in 2014 where it was discovered in Berks County, Pennsylvania (2). The insect quickly dispersed to multiple counties across Pennsylvania, and has now invaded New Jersey, Maryland, Delaware, Virginia, and West Virginia, with individual sightings reported in further surrounding states (1). While in its native range the insect does not represent a pest species, in the U.S. SLF has the potential to become a greater threat, because it is a generalist, a robust phloem feeder, it lacks natural enemies, and thus can reach high populations, in the hundreds, on single plants, if not controlled by insecticides (1). Though *Ailanthus altissima* is a preferred host of SLF, the insect can feed on other trees such as black walnut, maple, fruit trees, and grapevines (1).

Damage caused by SLF on grapevine can be extensive, if SLF establishes in a vineyard in high numbers (3) and if the insects are not managed, or if the insects migrate from the surrounding areas in a vineyard multiple times per season. Economic losses are mainly related to increased use of insecticide, which is the only method currently available to control SLF population. Often SLF congregate on single vines (3) and their feeding, if unchecked, can reduce photosynthesis, sap flow, carbohydrates such as starch, micro and macronutrients and amount of nitrogen in storage tissues. Heavy infestations of SLF on grapevines have been noted to reduce vine health by reducing carbon assimilation and increasing competition for important resources involved in plant growth and production (unpublished data). Furthermore, high density of SLF on vines in the previous season can reduce the number of clusters per shoot the following spring and may reduce vine hardiness and increase winter injury susceptibility (https://extension.psu.edu/spotted-lanternfly-management-in-vineyards). At this point, nothing is known about the molecular mechanisms governing the impact of SLF on grapevines or other plants, or the molecular responses of plants to SLF.

Aside from damage caused by the abundant ingestion of plant sap, SLF can also cause wounding to stems and trunks *via* its piercing stylet (4) and this damage can be magnified when inflicted by high number of SLF. Interestingly, SLF feeding is characterized by dark feeding lesions that can be observed by necked eye when pealing the bark of infested plants. Other phloem-feeding pests such as aphids, mealybugs, and whiteflies are much smaller-bodied than SLF and SLF size is much more like the one of the destructive glassy winged sharpshooter (*Homalodisca vitripennis*, Germar), that can pierce directly woody tissues but that feeds on plant xylem instead of phloem. While most of the damage caused by smaller piercing sucking insects is attributed to the consumption of photoassimilates and sometimes to their ability to vector pathogens but not to wounding (5), not much is known about the direct impact of SLF on plants while breaching the plant cell wall and physical barriers. The presence of dark lesions at feeding sites suggests that plants react to SLF wounding by promoting oxidation and production of secondary metabolites, as in other plant:insect interactions, and this hypothesis would need to be verified (6).

The voracious feeding and gregarious nature of SLF also causes copious amounts of honeydew to be excreted, leading to excessive sooty mold growth, that can also reduce plant photosynthesis and, in the long-term, vigor (3). Since many microorganisms can grow in honeydew and since insects are often associated to a multitude of microbes in their secretions (gut and frass), it cannot be excluded that plant responses to SLF can be also mediated by plant:microbe interactions (7–10).

The interactions of insects and their host plants are known to be specific to the organisms involved (6), thus, it is difficult to predict what impact an invasive species, such as SLF, will have in a certain system. Our understanding of how SLF and grapevines interact is still limited, but advances in this area might help explain why grapevines responses to SLF are not efficacious at repelling the insect and could help identify what plant defenses are employed by grapevines against SLF. Generally, plant responses may include a variety of defenses against insect stress, often including both active and passive defenses (11). Active defenses such as alterations in plant structure, secondary metabolite formation, and plant hormone responses can be monitored by analyzing the transcriptome and associated gene regulation under insect attack (11, 12). These plant responses vary across types of herbivorous insect feeding, with significant differences seen between chewing insects versus piercing and sucking phloem-feeding insects (12).

Feeding by either chewing or piercing and sucking insects can induce regulation of genes involved in plant defense-related processes and repress the expression of genes responsible for photosynthesis and plant development (12). However, differences exist in plant hormonal response, specifically between the generally antagonistic jasmonic acid (JA)/salicylic acid (SA) pathways (11, 12). Attack by chewing insects has been shown to repress the SA pathway and upregulate JA production, while phloem-feeders elicit the opposite (5, 12). This difference may be attributed to the contrast in physical damage to the plants, with phloem-feeders causing less overall damage (5, 12). It is also worth noting that while in most reported cases of phloem-feeding insect attacks pathogenesis-response transcripts, proteins, and/or activities are elevated, this response is not associated with chewing insects (5).

In addition to hormone regulation, attack by phloem-feeding insects may lead to alterations in plant structures. These changes include cell wall thickening, lignification, stomatal closure, and formation of a waxy cuticle (11). Structural changes are induced through a variety of defense mechanisms interacting with each other in different ways. For example, lignin production is associated with the oxidation of phenolic compounds by peroxidases, while peroxidases themselves are important enzymes involved in reactive oxygen species reduction (11). Plants have also been reported to respond to mealybugs and aphids with an increase in Ca^2+^ signaling and callose deposition to aid in repairing wounds and strengthening phloem cells by stomatal closure (5, 13).

While several studies have examined the effect of prominent phloem-feeding insects on a variety of plants at the transcriptomic level, there have been limited studies on the response of grapevines to phloem-feeding insects. In addition, in grapevines there has been reported a wide variation in plant responses dependent on the insect/host relationship, with specificity as narrow as plant variety (14). The present study aims to examine effect of prolonged SLF feeding on a *Vitis inter-specific hybrid* ‘Marquette’, at a transcriptomic level, and to elucidate some of the mechanisms responsible for the detrimental effect reported in SLF infested vineyards.

## 2 Materials and methods

### 2.1 Plant material and experimental design

The study was conducted at the Penn State Berks Campus (Reading, Pennsylvania, USA; 40.364702° N, 75.976374° W) located in southeast Pennsylvania. The experimental material was twelve 6-year-old hybrid ‘Marquette’ vines grown on a custom-made substrate (field topsoil, perlite, and peatmoss mixed at a 1:1:1 proportion, and pH kept at 7.1) in 38L plastic pots. Pots were painted white to reduce radiative heating from growing under outdoor conditions. The pots were arranged in two parallel rows of six vines in each row. A completely randomized design was used to assign half of the vines (six) to a control treatment and the remaining six to an adult SLF treatment. All vines were covered with an insect barrier netting bag with zippers (1.3 m × 1.4 m, AgFabric, WellCo Industries, Inc., Corona, California, USA) to avoid SLF escape and entrance. Eighty adult SLF, collected from nearby woodlands were released inside each netting bag on vines assigned to SLF treatment. SLF were kept on the vines from August 19^th^ through September 30^th^. Vines were monitored three times each week, and dead insects were counted and replaced with live ones. At the end of the experiment stem tissue which developed during the growing season (i.e., canes) was harvested from all vines and 10-15, 5 cm long cane pieces were randomly sampled from all areas of each vine to make a composite representative sample for each vine. These cane pieces were put in plastic Ziplock bags and transported to the laboratory (University Park, Pennsylvania, USA, 40.7982° N, 77.8599° W) inside coolers filled with dry ice. Upon arrival, the cane pieces were flash frozen in liquid nitrogen and immediately stored in a freezer at -80°C.

### 2.2 Sample processing and RNA extraction and quality

Cane pieces, stored in -80°C freezer, were used for extracting RNA. Sample bags containing cane pieces were taken out of the freezer, put on dry ice and peeled of their lignified outer bark to expose the green phloem tissue underneath. The phloem tissue was rapidly scraped off into a pre-chilled mortar and pestle. The scraped tissue was hand-ground into fine powder by pouring liquid nitrogen into the mortar and grinding using a pestle. About 50 mg of ground tissue was homogenized in a cetyltrimethylammonium bromide (CTAB) based buffer with a chloroform denaturation step and the RNA was selectively precipitated with LiCl following Blanco-Ulate et al. (15). RNA was cleaned up using a RNeasy Plant Mini Kit (Qiagen Sciences Inc, Germantown, Maryland, USA) including the DNase treatment on column. Purity of extracted RNA was measured with a Nanodrop 2000 spectrophotometer (Thermo Fisher Scientific, Waltham, Massachusetts, USA) and Bioanalyzer (Agilent 2100 Bioanalyzer system, Agilent Technologies, Santa Clara, California, USA).

### 2.3 RNA sequencing, mapping and annotation

Extracted RNA was sent to the Genomics Core Facility of the Huck Institute of the Life Sciences at Penn State for sequencing where a unique dual indexed library was prepared from each sample using the TruSeq Stranded mRNA kit according to the manufacturer’s instructions (Illumina, Inc., San Diego, California, USA). The concentration of each library was measured, and an equimolar pool of the libraries was made using the KAPA Library Quantification Kit Illumina Platforms (Kapa Biosystems, Inc., Wilmington, Massachusetts, USA). The library pool was sequenced using a NextSeq 550 High Output 75 nt single read sequencing run. Raw reads are deposited to NCBI under the BioProject accession no. PRJNA860209. This provided an average of ~58 million reads per sample. Sequences were then analyzed through a series of bioinformatics tools using Unix commands and R. In summary, the quality of the raw reads for all samples provided by the sequencing facility, were preprocessed and checked using Fastqc (16). Hisat2 (17) was used to align and assemble the sequences against the reference genome the *Vitis vinifera* (PN40024) genome assembly 12X.v2. Mapped sequences were then annotated using the *Vitis vinifera* VCost.v3 annotation version.

### 2.4 Differential gene expression (DGE) and gene enrichment analysis

Reads for the annotated genes per sample were counted by featureCounts (18). Finally, differential gene expression (DGE) patterns across treatments were analyzed by using the DESeq2 and edgeR package in the Bioconductor library (19).

Significant DEGs in the treatments were functionally characterized by using the annotation described in the Plant and Fungi data integration database (Grapevine reference genome assembly). However, due to the limited Gene Ontology (GO) information in the grapevine genome, we used grapevine gene IDs to find the best match ortholog genes (TAIR IDs) in *Arabidopsis thaliana* as described in the same database. To crosscheck and validate, reciprocal blast was also performed using orthology package in R that implements gene orthology inference using the reciprocal best hit (RBH) method as described by Drost et al. (20). These IDs were then used to conduct gene enrichment analysis using DAVID bioinformatics resources v6.8 (21).

## 3 Results

### 3.1 Sequence mapping and reads assembly

**Figure 1:**
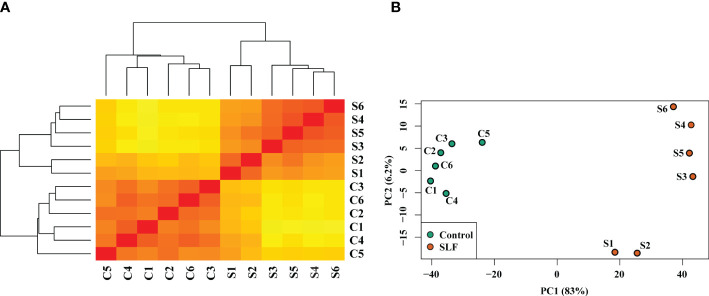
Sample distance matrix and principal component analysis of the treatments and replicates. **(A)** Dendrogram and sample distance matrix among the samples. Replicates for both C and S were clustered together and separated by treatment. Here red and yellow colors indicate, respectively, the closely and distantly related samples based on the read counts of DEGs. **(B)** Principal component analysis plot of relative distribution of the biological replicates and the treatments. PC1 (83%) and PC2 (6.2%) together explain approximately 90% variation of the samples.

Sequence mapping and percent genomic alignment are summarized in **Supplementary Table 1**. For both treatments (‘Marquette’ grapevines infested with SLF (S) and uninfested controls (C)), the overall alignment percentage was 86-91%. We estimated the distribution of samples by sample distance matrix (SDM) and principal component analysis (**Figures 1A, B**). We found strong clustering of biological replicates for control and for SLF treatments, except for replicates S1 and S2. An analysis of variation in the dataset using principal component analysis showed similar grouping of replicates for both treatments. The low percent of variation with PC2 (6.2%) indicated that although S1 and S2 were placed lower than the other replicates in the same treatment group they were not highly different considering PC1, which explained 83% of the variation. To test if inclusion of S1 and S2 in the analysis might affect the differential expression, we plotted and compared each replicate among the treatments using scatter plots (**Supplementary Figure 1**). These plots didn’t show any abnormal shape and distribution of gene expression for any pairwise comparison, so we considered all the replicates in the differential gene expression analysis.

### 3.2 Differential gene expression (DGE) analysis

**Figure 2:**
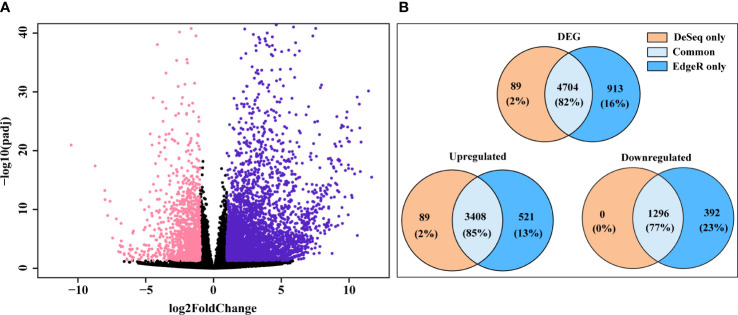
DEG found in DESeq2 and edgeR. **(A)** Volcano plot of significantly up and downregulated genes. X-axis and y-axis denote the Log2FoldChange and -log10 of padj values, respectively; where log2FoldChange ≥ 1.0 and padj < 0.05 were considered as significant and indicated in violet (upregulated) and pink color (downregulated). **(B)** Ven diagram of DEG, yielded from DESeq2 and edgeR, showing genes discovered by each analysis or genes found by both analyses.

We analyzed and assessed the variation of the read counts for each DEG between replicates by dispersion plot (**Supplementary Figure 2A**). Read counts for each gene were clustered around the ideal fitted line, with the dispersion decreasing as the means of the normalized reads count increases, indicating that the data was a good fit for the DGE analysis. Expression of the top 5000 genes based on their read counts (considering both treatments) was examined by hierarchical clustering heatmap (**Supplementary Figure 2B**). The majority of these top 5000 genes had higher signal ratios (Z-scores calculated from the read counts of each gene) in the SLF treatment, indicating that more upregulated genes were found in the SLF than control treatments. DESeq2 and edgeR were used to identify DGE filtering on Log2FoldChange ≥ 1.0 and padj < 0.05, (indicated in violet and pink color, respectively), and are shown as a Volcano plot (**Figure 2A**). DESeq2 analysis yielded a total of 4,793 significantly DEGs, among which 3,497 genes were upregulated and 1,296 were downregulated (**Supplementary Table 2**). EdgeR returned 5,617 significantly DEGs, of which 3,929 and 1,688 genes, respectively were up and down regulated. Comparing the genes identified from both analyses revealed that 4,704 genes (82%) were common, while 89 (2%) and 913 (16%) genes were found respectively by only DESeq2 and by only edgeR, respectively (**Figure 2B**). All the genes that were found downregulated in DESeq2, were also captured by edgeR. Since almost all the genes captured by DESeq2 were also found by edgeR, we proceeded with the gene list identified with DESeq2 for functional analysis.

### 3.3 Gene enrichment analysis of the DEGs

**Figure 3:**
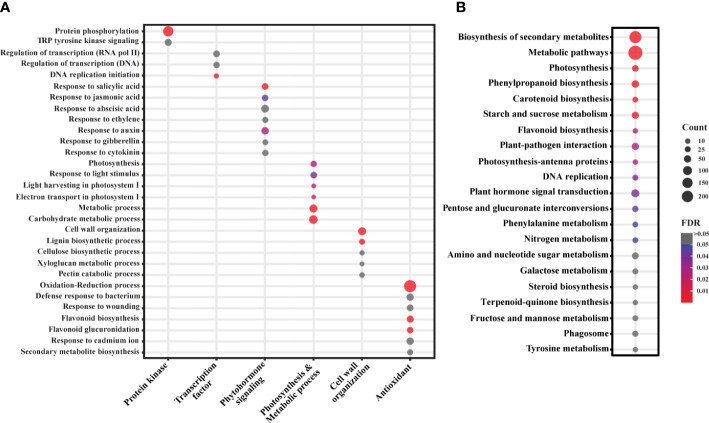
Gene enrichment analysis of the significant DEGs. **(A)** GO analysis of the significant DEGs. Selected BPs are categorized based on their functions in plants. **(B)** KEGG analysis of the DEGs. Color and bubble size indicate the false discovery rate (FDR) and the number of genes (count) belonging to each class, respectively.

We annotated the functions of the significant DEGs using the annotation described in the Plant and Fungi data integration database (Grapevine reference genome assembly). However, due to the limited Gene Ontology (GO) information in the grapevine genome, we used grapevine gene IDs to find the best match ortholog genes (TAIR IDs) in *Arabidopsis thaliana* as described in the same database. These IDs were then used to conduct gene enrichment analysis using DAVID bioinformatics resources v6.8., and the associated biological pathways (BPs), molecular functions (MFs), cellular components (CCs), and KEGG (KOs) pathways were retrieved. A total of 162 BPs, 91 MFs, 45 CCs, and 24 KEGG pathways were enriched with a False discovery rate (FDR) ranging from 3.0X10^-9^ to 0.9 (**Supplementary Table 3**). Among these, we found 33 BPs, 23 MFs, 31 CCs, and 15 KEGGs enriched with FDR < 0.05, which can be considered as the most probable pathways triggered by SLF infestation (**Supplementary Table 3**). Pathways were manually curated and sorted out the prospective biological pathways and KEGGs for a more comprehensive analysis (**Figures 3A, B**). BPs were grouped by their generic functions and assigned into major functional categories such as protein kinase, transcription factor, phytohormone signaling, photosynthesis and metabolic process, cell wall organization, and antioxidant (**Figure 3A**).

### 3.4 Analysis of DEGs elicited by SLF infestation

The aggressive group feeding nature of SLF can lead to wounding, which in turn may trigger plant defense responses and signaling involved in maintaining physiological homeostasis. However, effective host plant responses depend on the specific insect-plant interactions and how the plant perceives and orchestrates these signals. Therefore, in this study, we focused on the pathways related to insect-plant interactions, their signaling, host responses, and cellular homeostasis.

#### 3.4.1 DEGs involved in insect-plant interactions and signal transduction

##### 3.4.1.1 Signaling kinases

Plant responses to an insect begin with the recognition of plant-insect interplays occurring during the feeding time, such as the diverse mechanisms induced by oral secretions. For instance, herbivore-associated molecular patterns (HAMPs) could be recognized by plant cell wall receptors, resulting in the activation of signal transduction cascades carried by the secondary messenger molecules, such as cyclic AMP, cyclic GMP, inositol triphosphate, diacylglycerol, calcium, etc. In most cases, signal cascades start with the phosphorylation of related proteins mediated by protein kinases. 257 and 26 genes up and downregulated, respectively, related to protein phosphorylation (**Supplementary Table 4**). Among these, many signaling kinase genes, such as *LRR receptor kinase*, *LRR transmembrane protein kinase*, *NBS-LRR receptor kinase*, *S-locus protein kinase*, *Serine/Threonine receptor-like kinase*, *wall associated kinase*, and *FLG22-induced receptor-like kinase* showed enhanced expression under SLF infestation. Stimulation of protein kinase genes like *FLG22-induced receptor-like kinase* suggests presence of microbes, either deposited by SLF or exogenous microbes mobilized in the wounds. Our data also suggests that interchanges of signals triggered by protein kinases consequently induces the expression of transcription factors (TFs) in the nucleus, followed by activation of other signaling molecules like phytohormones and secondary metabolites, with their downstream target genes responsible for defense and physiological functions such as detoxification and photosynthesis.

##### 3.4.1.2 Transcription factors regulation

TFs are the master regulators that control the expression of genes at transcriptional level under different physiological conditions. The past few decades have been productive in identifying the TFs that are involved in regulating diverse cellular functions. These TFs mostly belong to large gene families, and their regulatory networks often overlap and function together (22).

**Figure 4:**
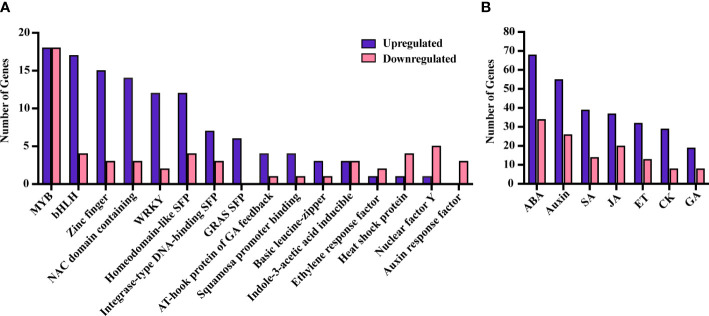
DEGs belonging to different TF families **(A)** and phytohormones **(B)**. SFP, super family protein.

**Figure 5:**
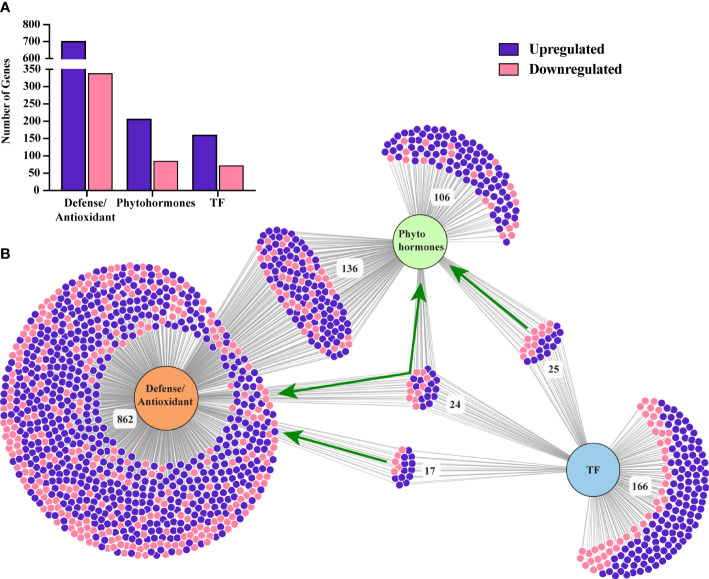
**(A)** Number of differentially expressed genes belong to plant defense, phytohormone signaling, and TFs. **(B)** A visualization of a number of common and distinct genes related to these three categories. Green arrows indicate the TFs related to plant defense, phytohormone and both. Figure was generated using a web-based visualization tool, DiVenn (26).

A total of 232 TFs, assigned to various functional categories/gene families, were differentially expressed in our data. Most (160) were upregulated under SLF infestation (**Supplementary Table 5**). TFs that are members of the *myb domain containing protein* family contained the highest number of DEGs (23) (**Figure 4A**). MYB proteins are one of the largest families of plant TFs that have been linked to many distinct functions, especially in regulating plant stress responses (22, 24). The other major TF families that have been associated with defense signaling are *basic helix-loop-helix* (bHLH), *ethylene-responsive-element-binding factors* (ERF), WRKY families, *NAC domain containing proteins* (NACs), *basic leucine-zipper (bZIP)*, and *zinc finger* (25). Each of these TFs were detected in our study, with most of them upregulated (**Figure 4A**). TFs involved in plant defense (17), phytohormones regulation (25), and both (24) were also differentially expressed (**Figure 5** and **Supplementary Table 5**).

##### 3.4.1.3 Phytohormone signaling

Phytohormones are small signaling molecules that are essential for the regulation of plant growth and development, and are deployed by plants as a universal strategy to defend against stresses (27, 28). It is well documented that SA and JAs, along with abscisic acid (ABA) and ethylene (ET), carry the major primary signals in modulating a wide range of adaptive immunity under stress conditions (27). However, more recently, the crucial roles of auxins and other phytohormones under stress conditions have also been reported (27). The direct involvement of plant growth regulators in plant defense suggests that the regulation of plant growth, development, and defense are intertwined and are part of a complex regulatory circuits of cross-communicating hormone signaling pathways.

Genes related to all the major phytohormones were enriched in our study, with 206 and 85 unique genes up and downregulated, respectively, under SLF infestation (**Supplementary Table 6**). Among these, we found 136 genes were directly involved in plant defense (**Figure 5**; **Supplementary Table 6**).

Genes responsible for ABA signaling were highly enriched in the dataset, with 68 and 34 genes up and downregulated, respectively (**Supplementary Table 6** and **Figure 4B**). ABA is commonly associated with plant growth and acts as a major regulator in abiotic stresses, however its involvement in biotic stresses is becoming more evident (29, 30). For instance, ABA both acts synergistically with JA under wounding or herbivorous insect attack, while also affecting resistance against necrotrophic pathogens (31, 32). Multiple copies of genes related to ABA, abiotic stress, and diverse cellular activities were upregulated in our study including BURP domain-containing protein (RD22), DREB2C, aquaporins, annexin 4, phospholipase D alpha and others (33–40). We also found differential regulation of several genes belonging to the ABC transporter G and B families which are necessary for wax transport to the cuticle and detoxification of xenobiotics (41, 42).

While SA, JAs and ET are naturally expected as these hormones are the primary regulators of inducible defenses, our data suggests inconclusive roles of these phytohormones under SLF infestation (**Supplementary Table 6**). However, we found that auxin biosynthesis and signaling related genes were the second highest enriched class of genes (**Figure 4B** and **Supplementary Table 6**). Auxin is associated primarily with plant growth and development, but also plays roles in plant defense *via* utilizing the secondary metabolite and TFs regulatory network. Thus, our data on phytohormones suggest that grapevines invest simultaneously on defense and in cellular homeostasis.

#### 3.4.2 DEGs involved in cellular homeostasis and host responses or resource reallocation

##### 3.4.2.1 Photosynthesis

Photosynthesis is part of the primary metabolic processes in plants and is a key indicator of their physiological condition. A total of 84 genes related to photosynthetic processes were differentially expressed under SLF feeding pressure (**Supplementary Table 7**). Among these, 77 genes were upregulated, with only 7 genes downregulated. We observed a strong upregulation of genes related to PSI reaction center subunits, PSII, phototropic-responsive NPH3, Rubisco, and light-harvesting chlorophyll binding (LHCB) proteins under SLF feeding pressure.

##### 3.4.2.2 Cell wall reformation and stomatal closure

Our data suggest that cell wall reformation and stomatal closure are two other crucial events that may take place under SLF infestation. Genes that are reportedly involved in stem lignification, such as peroxidases and laccase (43) were enriched in our analysis (**Supplementary Table 8**). In addition, lignins, which are complex cell wall polymers, are produced by the oxidative polymerization of monolignols in assistance with plant oxidases, peroxidases, and/or laccases (44, 45). Out of 19 peroxidase and 19 laccase DEGs in grapevine, 13 and 12, respectively, were upregulated (**Supplementary Table 8**). Furthermore, we found enrichment of genes involved in the biosynthesis of major structural components of the cell-wall matrix and its organization. For instance, genes responsible for the formation of cellulose, xyloglucan, and pectin were significantly upregulated upon SLF feeding (**Supplementary Table 9**). This results a role for stimulation of cell wall reformation pathways under SLF infestation in grapevine.

Additionally, 76 genes that are categorized as ‘response to cadmium ion’ (**Supplementary Table 10**) were differentially expressed. Genes responsive to cadmium ion or any heavy metals induce callose deposition in the cell wall, which in turn may stimulate stomatal closure (46). Additionally, insect herbivores feeding on the vascular system can induce hormonal responses resulting in stomatal closure (47, 48). Differential expression of genes such as glutamate receptor (GLR) proteins and receptor kinases that are involved in stomatal regulation indicate the plants’ promotion of stomatal closure as a response to SLF feeding (49–52).

##### 3.4.2.3 Plant defense and detoxification

Our data showed that SLF infestation triggered defense responses in grapevine by inducing multiple defense pathways recognized for biotic and abiotic stresses. A total of 1039 unique DEGs responsible for abiotic and biotic stresses and parts of a plant’s physiological immunity were assigned to defense/antioxidant category (**Supplementary Table 8**). The highest number of DEGs (363) belong to the oxidation-reduction process, where 263 and 100 genes were up and downregulated, respectively, under SLF infestation (**Supplementary Table 8**). Among them, the highest number of DEGs belong to the cytochrome P450 superfamily. These enzymes play a crucial role in detoxification of xenobiotics across animals, plants, insects, and microorganisms (53). Several *flavin-containing monooxygenase* and *glutathione S-transferase* DEGs that are involved in detoxification of toxic substances (54, 55) were also enriched in our data. Additionally, the upregulation (98 out of 120) of genes like *flavonoid 3’-monooxygenases, flavonone-3’-hydroxylase, flavonoid-3’-hydroxylase*, *flavonoid-3’,5’-hydroxylase, UDP-glucose:flavonoid 7-O-glucosyltransferase, flavonol synthase, chalcone synthase, stilbene synthase*, etc. support the idea of antioxidant pathway stimulation under SLF infestation (56).

### 3.5 Comparative transcriptomes analysis of grapevine varieties infested with similar pests

**Figure 6:**
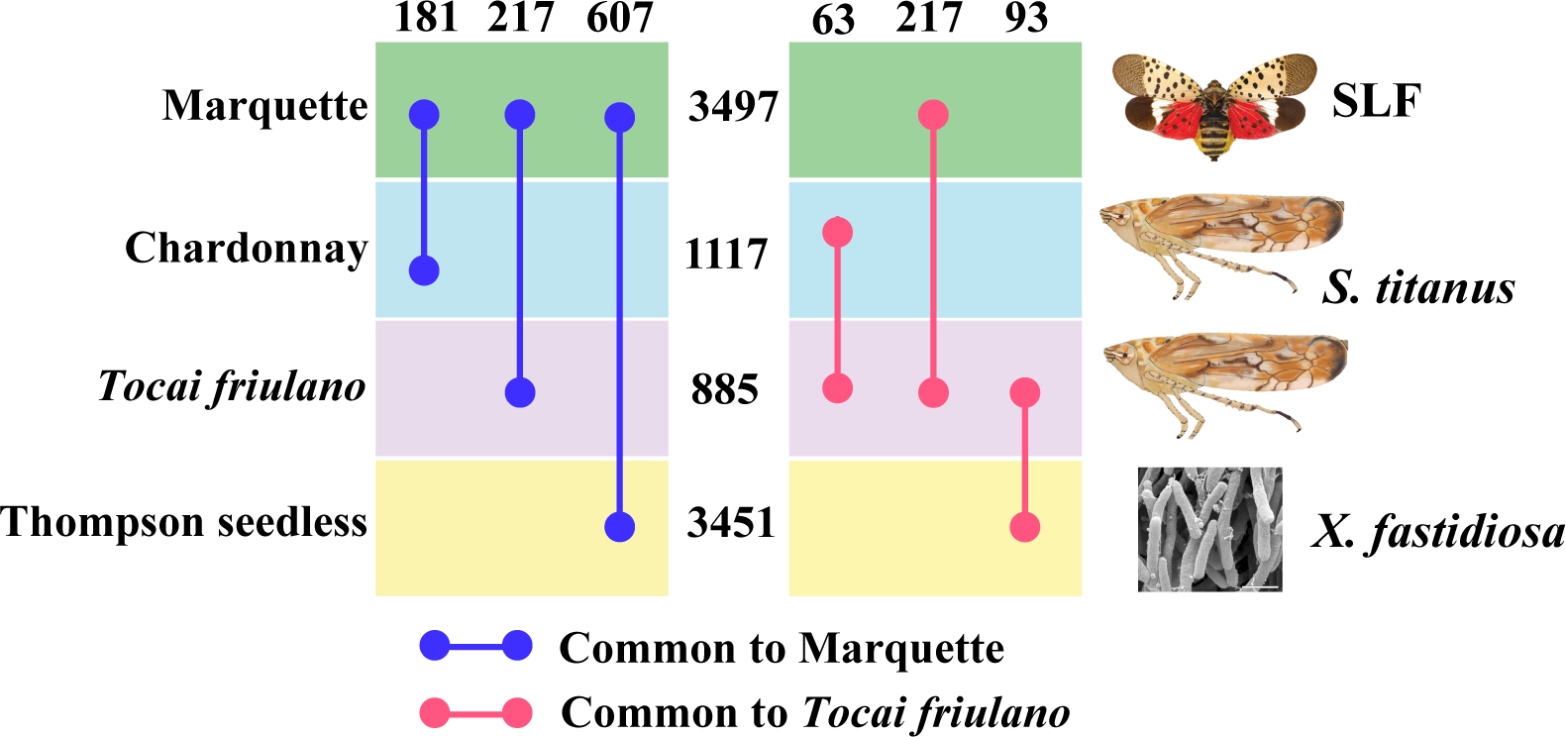
Comparative analysis of transcriptomes of different grapevine varieties under insect infestation and disease conditions. Numbers in-between horizontal boxes and on top indicate, respectively the total number of upregulated genes and the common upregulated genes between two varieties.

To put our results in the context of grapevine responses, we looked at other studies where grapevine was subjected to stress by insects similar to the SLF or by pathogens transmitted by similar insects. Surprisingly, not many transcriptomes that follow one of these two criteria have been published. We thus conducted a comparative analysis of transcriptomes using data from Bertazzon et al. (14) and Zaini et al. (57). Since there were not many common downregulated genes among the studies, we decided to conduct analysis only on the upregulated ones. Bertazzon et al. did a transcriptomic profiling on two grapevine varieties (Chardonnay and *Tocai friulano*) with different levels of susceptibility, former being the most susceptible to *Flavescence dorée*. This is one the most severe grapevine yellows diseases in Europe that is caused by phytoplasmas and transmitted by the leafhopper, *Scaphoideus titanus.* Authors carried out a comparative transcriptome analysis of both grapevine varieties in presence and absence of the vector and/or phytoplasmas. We used their data to sort out the genes that were significantly upregulated under insect infestation in both varieties (**Figure 6**). Our study on Marquette found a total of 3497 upregulated genes under SLF infestation, whereas Chardonnay and *Tocai friulano* had, respectively 1117 and 885 genes upregulated under leafhopper infestation (**Figure 6**). Among these, Marquette shared 181 and 217 common genes, respectively, with Chardonnay and *Tocai friulano*. On the other hand, Zaini et al. conducted a transcriptome analysis on grapevine var Thomson seedless, a susceptible variety to *Xylella fastidiosa*, the causal agent of Pierce’s disease of grapevine under disease and control conditions. *X. fastidiosa* is a bacterium transmitted by leafhoppers and sharshooters, but the study did not involve insects. Authors found a total of 3451 upregulated genes under disease condition, among which, 607 genes were common to our study (**Figure 6**).

We then analyzed the biological pathways of the genes shared among these grapevine varieties, which showed that Marquette, Chardonnay, and Thomson seedless plants triggered more defense pathways related genes than *Tocai friulano* (**Supplementary Table 11**). Since *Tocai friulano* is a relatively less susceptible variety, we also looked for genes which are common to this variety and unique, to comprehend genes or pathways that could be related to tolerance. *Tocai friulano* shared 217, 63, and 93 genes, respectively with Marquette, Chardonnay, and Thomson seedless, whereas 510 unique genes that were upregulated under insect infestation and could constitute genes for tolerance (**Figure 6**). A more in depth and investigative study of these genes in the future will help unveiling the mechanism of tolerance in the grapevine against insect infestation. Our comparative analysis also suggests that susceptible varieties tend to allocate more resources than tolerant varieties, when challenged by insects feeding, suggesting that a reallocation of resources could be detrimental to grapevines, if it would divert resources from the regular metabolic pathways. Further studies would be needed to explore this possibility.

## 4 Discussion

Spotted lanternfly is a phloem feeding insect that uses piercing and sucking to feed on the stem and trunk of host plants (4). On infested grapevines, over 100 adult SLFs can be clustered on a single vine. The aggressive and group feeding nature of SLF can cause a depletion of plant resources and consequently may increase susceptibility to pathogen invasion (4). Given the circumstances, understanding how grapevines respond to ‘heavy’ attack by SLF at the transcriptional level will advance our knowledge on how SLF interacts and impacts the host plant. To do so, we compared comprehensive, genome-wide transcriptional changes in SLF-free and SLF-infested ‘Marquette’ grapevines. We decided to test the gene expression level after long term feeding since the SLF effect are noticeable only in the season following the prolonged feeding event. RNASeq data generated from phloem tissue after one and half months of SLF infestation suggests that grapevine simultaneously induces defense and maintains cellular homeostasis *via* signaling cascades initiated by protein kinases, TFs, and phytohormones.

Plant defenses consist of structural barriers such as wax, lignin, and cuticle, and immune responses that induce active or adaptive immunity under adverse conditions (11, 58). We found plant-pathogen interaction, protein phosphorylation, TFs, and plant hormone signal transduction were enriched according to GO categories and KEGG pathways analysis (22, 27). These pathways control the plant’s physiological homeostasis and regulate the active defense response under stressors. The active defense response is a fine-tuned co-regulation of complex interchanges of signals triggered by plant-pathogen interactions orchestrated by series of signaling molecules like protein kinases, phytohormones, TFs, and activation of their downstream target genes. Many genes belonging to the categories of protein kinase, TF, and phytohormones were significantly expressed in our data. To categorize the differentially expressed TFs based on their functions we found that 66 out of 232 TFs were involved in plant defense and phytohormones regulation, whereas the rest may be involved in other physiological pathways.

Our results on phytohormone genes showed a rather noteworthy phenomenon. It has been reported that chewing herbivores are largely associated with the JA-mediated response, while phloem-feeding insects, such as SLF, are often associated with the SA-mediated response and a somewhat weaker JA response (59–61). However, we observed that similar number of genes from both pathways were induced by SLF feeding. Most of them were defense related TFs with a few downstream and signaling pathways related genes, such as PR-1 and LOX precursor 1. Therefore, SLF induced defense signaling connecting to SA or JA mediated pathways was inconclusive from our data.

Remodeling of the plant cell wall is a frequently reported phenomenon against pathogens or herbivores (62, 63) and is often associated with cell wall reinforcement (64) or the release of signaling molecules from the cell wall (65). KEGG pathways analysis of DEGs showed that biosynthesis of secondary metabolites, phenylpropanoid biosynthesis, phenylalanine metabolism, and flavonoid biosynthesis were enriched by SLF infestation. The biosynthesis of phenylpropanoids begins with the conversion of phenylalanine to cinnamic acid by phenyl ammonia-lyase (PAL), leading to the formation of different forms of phenolics, including lignin (66). Enhanced expression of genes in the general phenylpropanoid pathway such as PAL, 4CL, C4H, peroxidase, and CCoAOMT strongly infer the stimulation of lignin biosynthesis under SLF feeding. We have also found peroxidases and laccase genes that reportedly function in lignification were enriched in our data (43–45) supporting the hypothesis of structural defense upregulation in response to SLF. Lignin plays a crucial role in plant defense against herbivores by physically restricting the entry of insects through increasing the robustness of cell wall. It also decreases the nutritional content in the area, thus reducing feeding by the herbivores (11). Additionally, we found upregulation of genes that are involved in the biosynthesis of the major structural components of cell-wall matrix and their organization, such as cellulose, xyloglucan, and pectin. We also observed DEGs responsible for callose deposition which may eventually stimulate stomatal closure. Plants regulate stomatal closure as a strategy for cell wall strengthening, as well as maintaining photosynthetic rate (46). This is one of the key adaptive response of plants against herbivores (67). Several insects use stomatal openings for feeding sites (68–70) and oviposition (71). Oral secretion from insects can induce herbivore-associated molecular patterns (HAMPs) that could result in stomatal closure (67). Moreover, insect herbivores feeding on the vascular system can induce hormonal response resulting in stomatal closure (47, 48). Differential expression of genes such as glutamate receptor (GLR) proteins and receptor kinases that are involved in stomatal regulation indicate the plants’ promotion of stomatal immunity as a response to SLF feeding (49–52).

In this study, we found a significant upregulation of DEGs involved in photosystem I and II, such as phototropic-responsive NPH3, precursors for chlorophyll pigment synthesis, ferredoxin, and enzymes involved in photosynthesis such as RuBisco and LHCB. An increase in photosynthesis related genes could be the result of the plant’s strategy to maintain physiological homeostasis, a result of SLF sequestering large amounts of photosynthates, or it could be related to the increased demand for components of the cell wall.

Furthermore, all the major classes of DEGs in oxidoreductase families were enriched in our data, with the highest number of genes belonging to cytochrome P450 superfamily. These enzymes play a crucial role in detoxification and also protect plants by enhancing antioxidant activity (53, 72, 73). Enrichment of flavin-containing monooxygenase and glutathione S-transferase genes also suggests these activities under SLF feeding.

**Figure 7:**
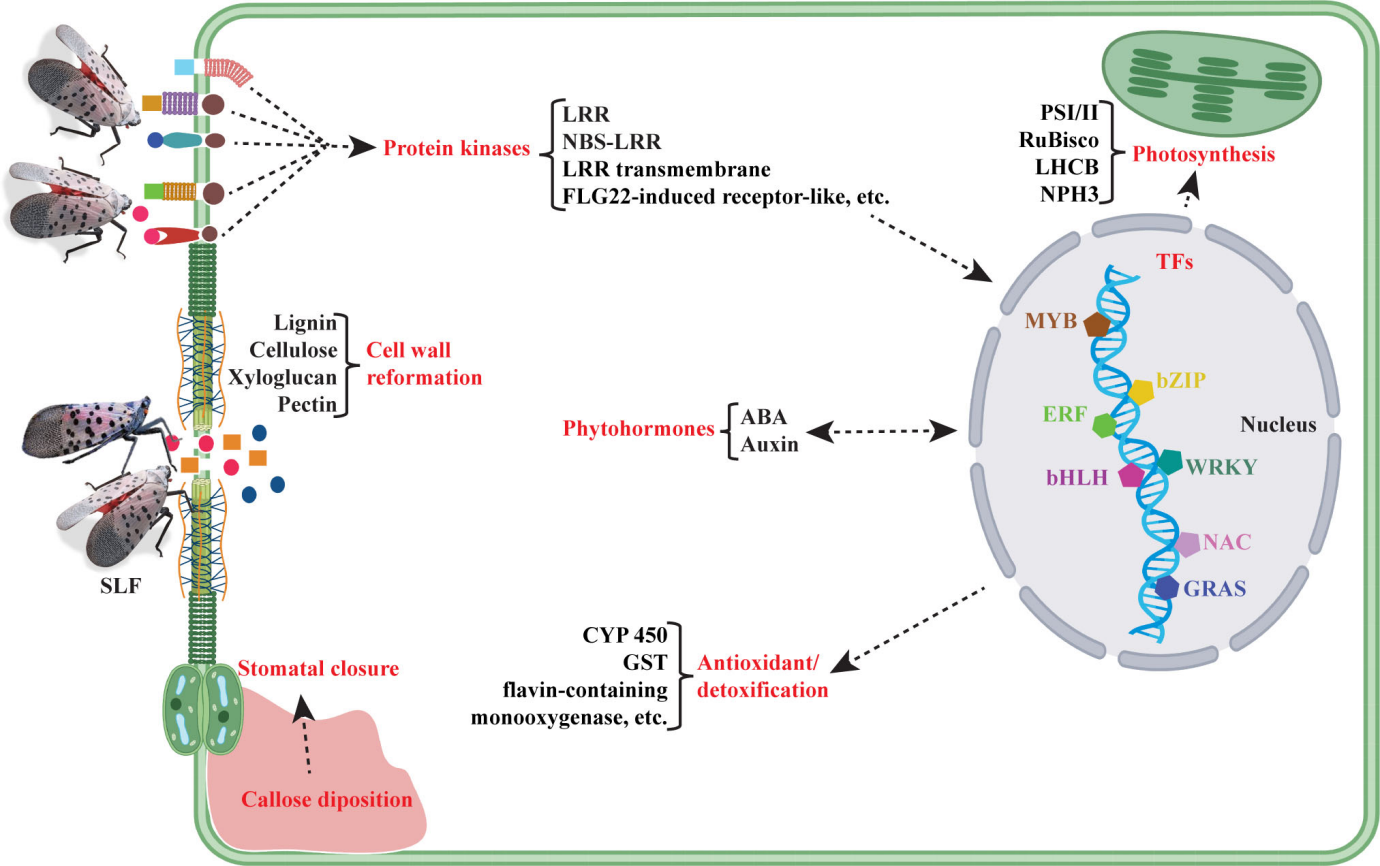
Molecular model of events occurring in hybrid *Vitis vinifera* ‘Marquette’ under SLF infestation, based on transcriptomic data. Here red color text indicates the categories of molecules or crucial events that are possibly happening upon SLF feeding, whereas black color text denotes the essential genes or pathways.

To summarize the complex and intertwining patterns of gene expression, we constructed a molecular model of events that may happen under SLF infestation (**Figure 7**). This study suggests that interactions between SLF and grapevines activate signaling molecules like protein kinases, TFs, and phytohormones. These in turn activate the downstream target genes responsible for various metabolic functions and defense, such as photosynthesis, cell wall reformation, stomata closure, and antioxidation/detoxification.

In conclusion, we conducted an experiment to evaluate the transcriptional response of heavy infestation of SLF on grapevine. Extensive changes in gene expression, particularly in pathways associated with biosynthesis of lignin and other structural components of cell-wall matrix, and antioxidant/detoxification indicate that grapevine likely responds to SLF feeding through remodeling of cell-wall and detoxification. Patterns of SA and JA response indicate that SLF attack elicits novel pathway interactions and suggests that future studies should explore more regarding the phytohormone signaling. We also carried out comparative transcriptomes analysis of grapevine varieties infested with similar pests. Our analysis suggests that under insect infestation, susceptible varieties tend to allocate more resources than tolerant varieties. Reallocation of resources, especially channeling off resources from the regular metabolic pathways, consequently, might be detrimental to grapevines.

## Data availability statement

The datasets presented in this study can be found in online repositories. The names of the repository/repositories and accession number(s) can be found below: https://www.ncbi.nlm.nih.gov/ PRJNA860209.

## Author contributions

MC and CR designed experiment. SK and CK-W performed RNA extraction. MI conceptualized data curation and analysis pipelines. MI performed data analysis and generated figures. MI, CK-W, and SK prepared original draft. MC, CR, JL, and MI wrote, reviewed, and edited manuscript. CR and MC supervised the study. All authors contributed to the article and approved the submitted version.

## Funding

This project was supported by the Pennsylvania Department of Agriculture under Agreement Number 44144949, the United States Department of Agriculture (USDA) National Institute of Food and Agriculture (NIFA) Specialty Crop Research Initiative CAP Award number 2019-51181-30014, and the USDA NIDA Federal Appropriation under Project PEN04628 (Accession number 1014131) and Project PEN04652 (Accession number 1016243).

## Acknowledgments

We are grateful to Lauran Briggs and John Rost for their technical assistance with SLF introductions and tissues collections and for maintaining the experimental vines.

## Conflict of interest

The authors declare that the research was conducted in the absence of any commercial or financial relationships that could be construed as a potential conflict of interest.

## Publisher’s note

All claims expressed in this article are solely those of the authors and do not necessarily represent those of their affiliated organizations, or those of the publisher, the editors and the reviewers. Any product that may be evaluated in this article, or claim that may be made by its manufacturer, is not guaranteed or endorsed by the publisher.

## Supplementary material

Supplementary Figure 1 Scatter plots for each replicate of the treatments **(A)**: Control, **(B)**: SLF. No replicate showed any abnormal distribution of gene expression for the pairwise comparison.

Supplementary Figure 2 Dispersion and hierarchical clustering heat map of each gene among the replicates. **(A)** Black dot and blue circle designate, respectively, the mean of normalized read counts and variation of a gene. Strongly clustered data points around the red line suggested that data were well distributed and fit for differential gene expression (DGE) analysis. **(B)** Hierarchical clustering heat map of differentially expressed genes among SLF (S) and control **(C)** treatments. Z-scores calculated from the read counts of each gene are shown in a blue-yellow color scale, where blue and yellow represent higher and lower read counts, respectively. Each column and row, respectively represents the replicates and a differentially expressed gene.
