# Supplementary figures and images for: Deciphering genome-wide transcriptomic changes in grapevines heavily infested by spotted lanternflies
Source: Front Insect Sci. 2022 Aug 25;2:971221. doi: 10.3389/finsc.2022.971221 (PMC10926465; doi:10.3389/finsc.2022.971221)

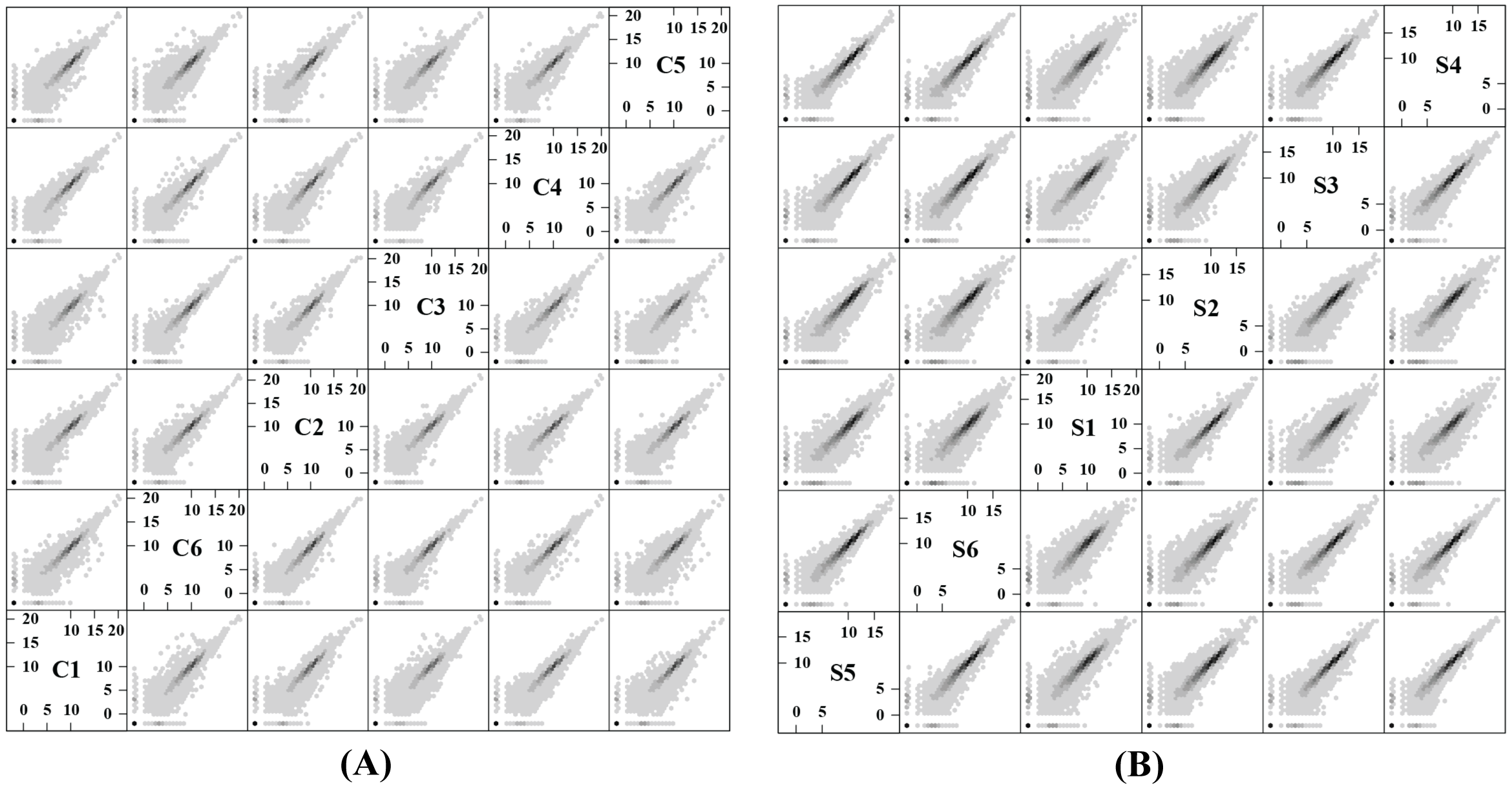

Supplement: Supplementary Figure 1 — Scatter plots for each replicate of the treatments (A): Control, (B): SLF. No replicate showed any abnormal distribution of gene expression for the pairwise comparison. [file DataSheet_1.zip › Supplementary Material/Supplementary Figure 1.tif]

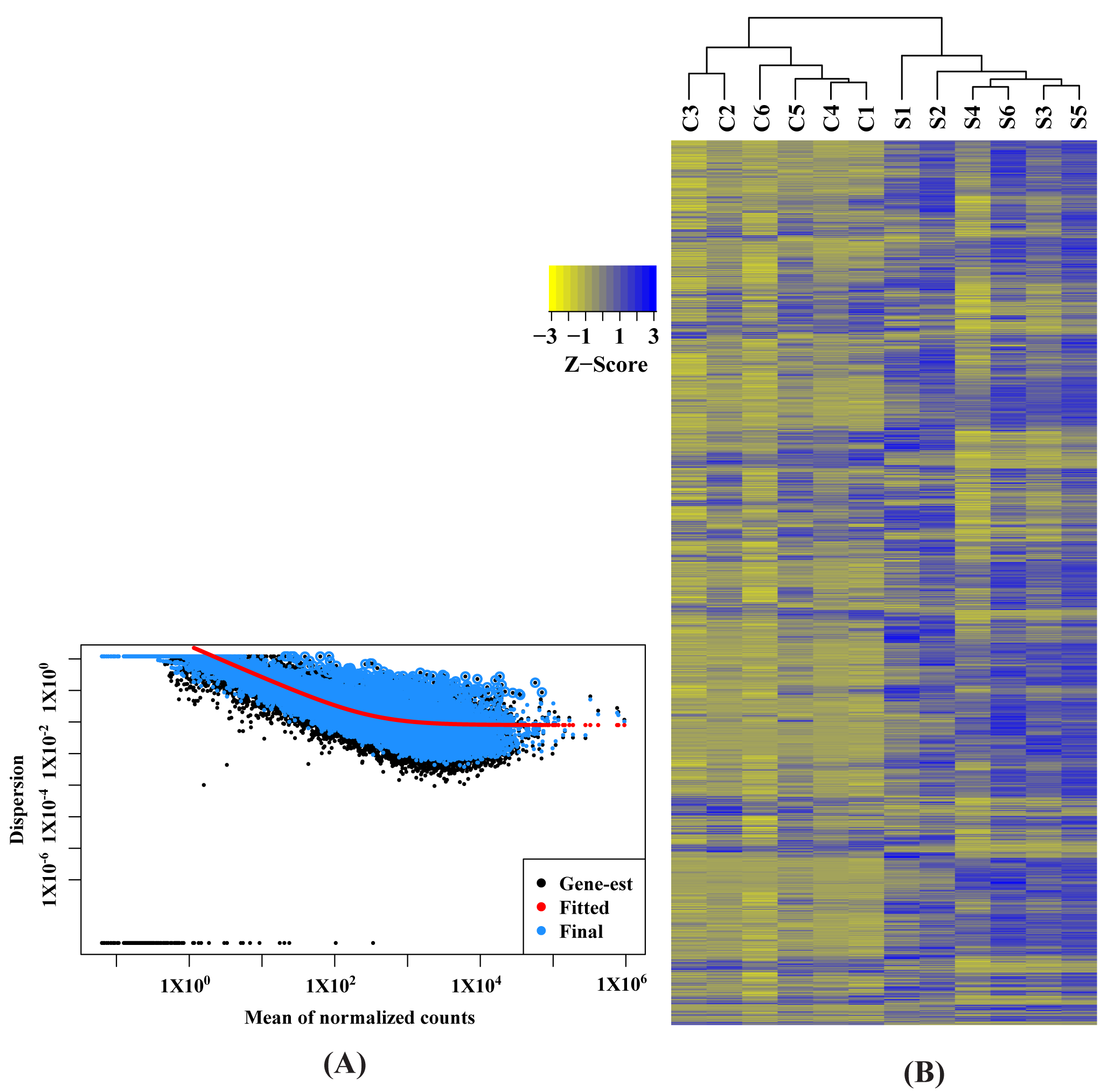

Supplement: Supplementary Figure 1 — Scatter plots for each replicate of the treatments (A): Control, (B): SLF. No replicate showed any abnormal distribution of gene expression for the pairwise comparison. [file DataSheet_1.zip › Supplementary Material/Supplementary Figure 2.tif]
